# Breaking down barriers to mental healthcare access in prison: a qualitative interview study with incarcerated males in Norway

**DOI:** 10.1186/s12888-024-05736-w

**Published:** 2024-04-17

**Authors:** Line Elisabeth Solbakken, Svein Bergvik, Rolf Wynn

**Affiliations:** 1 Department of Clinical Medicine, UiT The Arctic University of Norway, Tromsø, N-9038 Norway; 2 Division of Mental Health and Substance Use, University Hospital of North, Tromsø, Norway; 3 Department of Psychology, UiT The Arctic University of Norway, Tromsø, Norway; 4 Department of Education, ICT and Learning, Østfold University College, Tromsø, Norway

**Keywords:** Prison, Incarcerated, Correctional, Mental health, Help-seeking, Access, Healthcare, Utilization

## Abstract

**Background:**

Mental health problems are highly prevalent in prison populations. Incarcerated persons generally come from disadvantaged backgrounds and are living under extraordinary conditions while in prison. Their healthcare needs are complex compared to the general population. Studies have found that incarcerated individuals are reluctant to seek help and that they experience challenges in accessing mental healthcare services. To some extent, seeking treatment depends on the degree of fit between potential users and health services, and actual use might be a better indication of accessibility than the fact that services are available. This study aimed to explore individual and systemic facilitators and barriers to accessing mental healthcare in a prison context.

**Methods:**

An analytical approach drawing on elements of constructivist Grounded theory was the methodological basis of this study. Fifteen male participants were recruited from three prisons in Northern Norway. Data was collected through in-depth interviews on topics such as help-seeking experiences, perceived access to services and availability of health information.

**Results:**

We found that distrust in the system, challenges with the referral routines, worries about negative consequences, and perceived limited access to mental healthcare were barriers to help-seeking among incarcerated individuals. How prison officers, and healthcare personnel respond to incarcerated persons reporting mental distress could also be critical for their future willingness to seek help. Providing information about mental health and available services, initiating outreaching mental health services, and integrating mental health interventions into treatment programs are examples of efforts that might reduce barriers to accessing services.

**Conclusions:**

Facilitating access to mental health services is crucial to accommodate the mental health needs of those incarcerated. This study provides insights into the complex interplay of individual, social and systemic factors that may contribute to the utilization of mental health care among incarcerated persons. We suggest that correctional and healthcare systems review their practices to facilitate access to healthcare for people in prison.

## Background

### Mental health of people in prison

The rates of mental disorders are considerably higher among incarcerated individuals than in the general population [1–4]. Co-morbidities are common, and around 20% of incarcerated individuals have concurrent mental and substance use disorders [5]. They are at increased risk for all-cause mortality, self-harm, violence, and victimization, and suicide rates are about 3–6 times higher among incarcerated males relative to males in the broader population [6]. Adverse life experiences and disadvantaged living conditions from an early age may explain the observed accumulation of mental health problems in prison populations worldwide [7, 8]. Genetic predispositions combined with environmental stressors are implicated in the development of mental disorders [9, 10]. People in prison generally experience low educational achievements, low income, and unstable housing. Thus, the poor mental health of prison populations is caused by a complex interplay of social, environmental, and genetic factors [7, 11]. In addition to the pre-existing burdens, incarcerated individuals are facing prison–specific challenges such as loss of autonomy, social isolation, bullying and violence that may exacerbate mental health issues [12–15]. Considering the elevated rates of mental health problems in prison, facilitating access to mental health services is crucial to accommodate the needs of those incarcerated.

### Access to mental health services

The *treatment gap* refers to the proportion of individuals with mental health problems within a specific community that require treatment without receiving it [16]. Variable access to mental healthcare and high levels of unmet mental health needs are universal challenges in communities across the world [17, 18]. Even when health services are available, individual and systemic barriers may hinder their use. In a narrow sense, *access* to healthcare may be considered equivalent to available services. However, some argue that a more meaningful way to define access is the “degree of fit” between the potential users and health services [19]. For instance, if services are accessible in terms of transportation and treatment costs and whether they are compatible with potential users’ personal attitudes, beliefs and preferences. “Having access” can be understood as the potential for using available mental health services. “Gaining access”, is the individual process of choosing to use those services [20]. Within this frame of reference, access to services is more precisely defined by the actual use of services.

### Mental health help-seeking

Across settings and populations, the majority of those suffering from mental health problems do not seek treatment [21–23]. The literature on help-seeking gives insight into the intrapersonal factors involved in accessing mental health care. Within this context, help-seeking has been defined as: *" an adaptive coping process that is the attempt to obtain external assistance to deal with a mental health concern”* [24]. The process of seeking help involves becoming aware of a mental health problem that may require intervention; articulating the psychological challenges in a way that can be understood by others; awareness of help sources that are available and accessible; and a willingness to talk about the mental health problem to available help sources [25]. Throughout the help-seeking process, personal thoughts and feelings become increasingly interpersonal as an individual confides in and seeks support from others. It is not uncommon to share mental health concerns with informal sources of support such as friends and family prior to, or even instead of, seeking professional help [26]. Moreover, informal networks are found to facilitate but may also discourage professional formal help-seeking for mental health problems [27, 28].

The theory of planned behavior (TPB), a well-known model within behavior change research, may also provide a framework for understanding how personal attitudes and social influences are implicated in accessing healthcare. Subjective norms, attitudes, and perceived behavioral control are elements of TPB that are particularly important for understanding the help-seeking process [29]. In this context, subjective norms refer to a person’s beliefs about other peoples’ practice or approval of help-seeking and are related to expectations of social support in pursuing professional help. Attitudes refer to appraisals of seeking professional mental help as beneficial or harmful and a judgement of whether help-seeking would be constructive compared to alternative behaviors. Perceived behavioral control can be divided into self-efficacy (the confidence that one can seek help), and controllability (the extent of personal control in the help-seeking process). A recent review found that attitudes and perceived behavioral control predict help-seeking intentions across different population groups and cultures [30].

### Access to mental health services in prison

Equity is essential in healthcare to ensure that the health system meets the needs of different groups of people and individuals [20]. “The principle of equivalence” is a widely endorsed standard for healthcare in correctional settings [31]. This principle is laid down in the United Nations´ Nelson Mandela Rules. Rule number 24 states that: *“Prisoners should enjoy the same standards of health care that are available in the community, and should have access to necessary health-care services free of charge without discrimination on the grounds of their legal status”* [32] (p.8) However, some argue that equal standards are not sufficient to meet the complex needs of incarcerated individuals and that mental healthcare in prison must be more intensive and integrative than services provided in the community [33–35]. In reality there are several reports of shortcomings in the delivery of mental healthcare in prison in many countries across the world, as mental disorders in incarcerated persons are underdiagnosed and undertreated [6, 34]. Studies from Canada, the US, and the UK indicate that a significant proportion of incarcerated people with mental health problems have not received adequate treatment [36–39]. Suggested explanations for unmet needs are underfunding, failure in screening procedures and quality at reception, demand for more mental health knowledge among prison staff, and possible underrating of the severity of mental health problems by the prison administrations to reduce treatment costs [36–40]. Taken together these reports suggest that mental health services do not fit the complex needs of incarcerated persons in high-income countries. There is less knowledge about the situation in low- and middle-income countries. However, the elevated rates of mental disorders in these countries suggest that unmet needs among incarcerated persons are a widespread challenge [1].

### Mental health help-seeking in prison

Evidence suggests that the immense burden of mental disorders among people in prison is not matched by a proportional use of mental healthcare [41]. Several reports from various correctional settings have documented that incarcerated persons are reluctant to seek help for mental health problems [41]. Among the reported barriers to help-seeking in prison are confidentiality concerns [42], fear of stigma associated with a diagnosis [43], a preference for self-management or informal support [44], lack of knowledge of psychological services [42, 44] and distrust in the system [45]. In addition, systemic factors may influence access to healthcare in prison. The culture in all-male prisons typically demands that those imprisoned mask their vulnerabilities by adopting a tough and dominant demeanor [46]. Experiencing mental illness and receiving professional mental health treatment is also associated with an increased risk of victimization in incarcerated individuals [47].

Mental health literacy (MHL) is a concept that includes the knowledge and attitudes that influence how people manage their mental health needs [48]. Having sufficient knowledge and access to information about mental health and mental health services can be a prerequisite for seeking professional help [49]. For people living in the community, seeking online information and advice is an important strategy for gaining knowledge about how to cope with mental health challenges [50–52]. For security reasons, access to the Internet is typically severely limited for those imprisoned [53, 54]. Hence, this essential mental health information source is largely unavailable to them. Accordingly, incarcerated individuals are reliant on finding mental health information through information pamphlets, books, TV programs, newspapers or consulting healthcare professionals [55]. Some argue that limited access to online information and digital health services may have consequences for the well-being and successful rehabilitation of those incarcerated [53, 56, 57]. Thus, there are reasons to believe that restricted access to mental health information may also affect help-seeking and access to healthcare for incarcerated individuals.

### The rationale for the current study

Fostering health-promoting environments and adequate access to mental healthcare within prisons is a public health imperative increasingly acknowledged in the literature [33]. Moreover, the mental health of incarcerated persons is a matter of public safety since untreated severe mental disorders are associated with a higher risk of recidivism [58, 59]. People in prison retain their right to health services, and in principle, incarcerated persons *have* access to mental health services. A vital question, however, is how incarcerated persons experience *gaining* access and how this affects their actual use of services. Existing research on the provision of mental healthcare in prisons, particularly within a Scandinavian context, is sparse, leaving significant knowledge gaps. The question of access to health information for incarcerated persons is similarly understudied. This study aimed to investigate how incarcerated persons experience individual and systemic factors that facilitate or impede access to mental healthcare in prison.

## Method

### Ethics

The Helsinki Declaration of Medical Research involving human subjects and services laid the basis of the ethical considerations of this study [60]. The study was approved by the Data Protection Officer of the University Hospital of North Norway (No. 02415). The Norwegian Correctional system, which is responsible for the welfare of incarcerated individuals, approved of the study (Ref. 200900463-347). The Regional Health Research Ethics Committee concluded that the project was outside their mandate (Ref. 40,701).

The principles of voluntariness and informed consent are central to human subject research. Individuals in prison are considered vulnerable due to their restricted freedom and autonomy, poor health status, higher incidence of learning disabilities, and lower literacy levels. Consequently, additional precautions are required to ensure that research with incarcerated participants is conducted ethically [61]. User participation in designing research that includes vulnerable groups is crucial to achieving this objective [61, 62]. Measures in accordance with recommendations were taken to ensure consent information that is complete, relevant, and understandable [63]. A user representative from Way-Back, an organization that supports incarcerated persons with reentry to their communities, contributed to the project’s planning. The user representative provided input on information about the study, research questions, the interview guide and how to conduct the interviews. The input was used to tailor information and for conducting the interviews in accordance with the constraints of the prison contexts and the needs of the incarcerated individuals. The choice of whether to reimburse participants in prison studies is debated. Because of the relative deprivation of prison life, some argue that even small incentives could potentially result in undue influence for participation in research [64]. For this reason, we chose to abstain from offering reimbursement for the participants in this study.

### Study context

At any given time, about 3000 persons are serving a sentence in Norway, of which 5.6% are women and 26.2% are non-Norwegian citizens [65]. A recent study found that almost 60% of incarcerated persons in Norway had a diagnosed mental disorder, together with a 33% rise in the one-year prevalence of mental disorders between the years 2010–2019 [66]. Thus, the proportion of people with mental disorders entering prison has been increasing. In Norway, access to necessary healthcare is considered a basic human right and is legislated in the Patient’s Rights Act section 2 [67]. Healthcare is primarily tax-funded, with a nominal service fee and a relatively low cap on yearly individual costs [68]. Norway has committed to “the principle of equivalence” meaning that those imprisoned retain their right to healthcare equal to that of the general population [31]. Prison health services serve incarcerated persons with milder mental health problems and are accessible by self-referral through a paper-based request system. The prison health services can refer those who experience moderate to severe mental disorders to specialist mental health services, and treatment is often provided in prison by mental health professionals from local hospitals For people imprisoned in Norway, healthcare and medications are free of charge [69], eliminating one significant barrier to mental healthcare access [70]. Furthermore, as the municipalities and local hospitals provide health services - the importation of services promotes equity and that services are independent of the correctional system, thereby strengthening the rights of people in prison [71].

A study found that incarcerated persons in Norway were reluctant to seek help for mental health problems from prison health services unless they had concurrent sleep or substance use problems [72]. A survey by Bjørngaard et al. [73] found lower patient satisfaction with prison health services compared to people using community health services and that those with mental health problems were less satisfied compared to incarcerated patients with other health challenges. A survey representative of the Norwegian prison population found that 20% of incarcerated males sample reported that they had received mental health services, while 25% reported that they had been in need of mental health services in prison but had not received any [11]. More recent reports suggest that mental health services are insufficient to meet the needs of those imprisoned in Norway and that incarcerated individuals referred due to their severe mental illness may not be admitted to specialist services for in-patient assessment and treatment [74, 75]. These reports indicate that mental health services do not fit the complex needs of incarcerated persons in Norway and that there are potential obstacles in their access to mental healthcare.

### Study design

This study was underpinned by relativist epistemology which is based on the assumption of multiple individual realities that allow for different understandings of the same phenomenon [76]. The study design was suitable for exploring and explaining commonly experienced individual, social, cultural and structural factors that influence help-seeking and access to mental healthcare for incarcerated individuals. The study incorporates vital Grounded Theory (GT) components, including initial coding, categorizing data, constant comparative methods involving inductive and abductive reasoning, and memoing [77]. The use of theoretical sampling, which is rare in prison research due to ethical and practical constraints [78], was not employed in this study. Data collection concluded once additional data no longer contributed new insights or further elaborated the developed categories.

### Preconceptions

The first author, a clinical community psychologist and a PhD student, worked part-time as a prison officer for two years during her psychology education. This experience gave her an insider’s view of the correctional system, inevitably influencing her initial perceptions. Before conducting the interviews, she held a somewhat optimistic view of the correctional system’s capacity to support and enhance the mental health of those incarcerated. However, this perspective was challenged through the narratives of the study participants, who conveyed powerful personal accounts that highlighted substantial barriers to obtaining mental health services within the prison environment. The other two authors, serving as supervisors, are also researchers and mental health professionals with considerable clinical experience. Their diverse backgrounds contributed to a supervisory dynamic that adresssed the research topic’s complexities. Throughout the study, the authors engaged in a process of collaborative reflection, concerned with maintaining a balance between engaging with participant stories and sustaining a critical stance towards the data. These discussions were essential in helping the first author navigate an empathetic understanding of the participant’s experiences with the necessary analytical objectivity required for rigorous qualitative research.

### Participants and study settings

Fifteen males serving a prison sentence were recruited from three prisons in Northern Norway. Thirteen of the participants served a sentence at a high security level, while two served at lower security. The participants’ age ranged from the early twenties to the late sixties (M: 43.6 years). Two participants had other nationalities, while the rest were Norwegian citizens. Further details about the participants must be withheld to preserve their privacy. When citing individual participants, they are anonymized by using pseudonyms.

### Recruitment

Participants were recruited through posters in the prison ward that conveyed basic information, including the fact that the interviews were confidential and would be recorded. The posters encouraged those interested in participating to approach a contact person for more information. A prison officer, a social worker or a reintegration coordinator were assigned the role as contact persons in the selected prisons. Those who actively approached the contact person were given more comprehensive written information about the study. Requiring an active choice by incarcerated individuals was done to enhance their experience of self-determination and autonomy in their decision to participate. The contact person scheduled appointments with the participants, and the interviewer had no prior knowledge of the participants other than what they presented in the interviews. One potential participant cancelled the interview appointment due to health issues on the interview day and withdrew from the study.

### Interviews

The first author conducted face-to-face, in-depth interviews. The interviews took place in prison visitation rooms or in an office in the health wards. Before the interview, the participants were given information about the study and their rights as research participants and signed a written consent form. The interviewer was alone with the participants during the interview and had a personal alarm as a safety precaution. The interview guide covered topics on knowledge of mental health and available services, help-seeking experiences, and access to mental health information (sample questions provided in Table 1). The participants were asked open-ended questions and were invited to speak freely on these topics. Thus, the order and framing of questions varied depending on where they fit into the participants’ narratives. This allowed for following up on the participants’ experiences and may have given the participants an increased sense of control in the interview. The first author who conducted the interviews was attentive to signs of emotional discomfort in participants and avoided pressure on sensitive topics. After the interviews, the participants were encouraged to ask questions and comment on their experience and reminded of their right to withdraw from the study. Nearly all the participants expressed that the experience of participating in the study was positive and that they appreciated the chance to contribute to the research project.

**Table 1:** Sample questions from the interview guide

*Q1: If you were to find out more about mental disorders. Where could you get information?*
*Q2: Where can one seek support for mental health problems in prison?*
*Q3: How could one proceed to seek professional help for mental health problems?*

### Analysis

The first author transcribed the audio-recorded interviews in Norwegian, ensuring a verbatim account of the participants’ narratives. The initial eight interviews were transcribed before initiating data analysis. This early examination of the data facilitated a refinement of the interview guide, which was then applied to the subsequent seven interviews to deepen the inquiry. Data collection and analysis were concurrent as the study progressed from the ninth interview, which allowed for immediate integration of new data into the evolving analytical framework. The data was examined using the NVivo 12 software, which supported the systematic organization and analysis of the data. The data was analyzed line-by-line, searching for incidents in the form of recurring beliefs, actions, experiences, and explanations [79]. The constant comparison method was applied throughout the analysis. In the initial coding phase, incidents were compared to incidents, and through this process underlying recurring concepts and similarities were identified and assigned codes. Subsequently, codes were then compared to codes, and related codes were organized into conceptual categories, reflecting both common features and divergent viewpoints [77]. In the intermediate coding phase, the data was abstracted into categories which were compared to each other, and relationships between categories were developed and refined. The authors engaged in a collaborative and reflective dialogue throughout this process, meeting regularly to deliberate on preconceptions, the emerging categories and their interpretations. This dynamic exchange was informed by memos that captured analytical decisions, insights, and evolving interpretations, thus guiding the reflective process. In the last stage, advanced coding, a core category which binds the other categories and sub-categories together was developed. Through a collaborative process the categories were substantiated with representative quotes, which, upon completion of the analysis, were translated from Norwegian to English for inclusion in the report. This resulted in a nuanced understanding grounded in the participants’ experiences and the researchers’ interpretative lens.

## Results

The data analysis yielded four main categories illustrating the participants’ active engagement in identifying challenges and facilitators for mental healthcare access within the prison environment. The first category, “Mental health awareness,” captures how beliefs and knowledge concerning mental health were influenced by the experiences and constraints inherent to prison life, potentially affecting the pursuit of help and access to healthcare services. The second main category reveals how systemic sub-cultural values can obstruct healthcare access, whereas, on a personal level, fellow inmates served as vital support for obtaining mental health services. The third main category, “Access to mental health care,” examines how organizational and systemic barriers impede access to mental healthcare. The final main category, “Enhancing access to services,” delineates factors that lowered the bar for mental healthcare access. The core category, “Breaking down barriers,” encapsulates the dynamic interplay between incarcerated individuals and the contextual factors that influenced their ability and willingness to access mental healthcare in prison. This central theme also recognizes the collaborative effort between participants and researchers in identifying problem areas and solutions to mental healthcare access, thereby “breaking down barriers”. An outline of these categories is presented in Table 2.

**Table 2:** Overview of results

Core category	Main categories	Subcategories
**Breaking down barriers**	**Mental health awareness**	• An information void • Awareness of mental health issues
	**Social influences on help-seeking**	• Prison culture and mental health stigma • The role of peers in accessing mental health services
	**Access to mental healthcare**	• Self-referral and disempowerment • The perceived availability of mental healthcare • Prison officers’ role in mental healthcare and accessing services
	**Enhancing access to services**	• The perceived advantages of seeking professional help • Lowering the bar for accessing mental health services • Mental health support from different sources

### Mental health awareness

#### An information void

Seeking information can be an essential first step for recognizing symptoms of mental illness that may require intervention. Prior to imprisonment, visiting their general practitioner or using online search engines were the preferred methods for finding health information for the participants in this study. In prison, however, access to the Internet is severely limited:

*Where can we get information? We do not have access to computers or anything. So, I would have to call someone on the outside to get them to print articles and send them to me by post. So, no. We don’t know our rights, we don’t know about the services available to us, as a matter of fact we know very little. There’s an information void.* ***Stuart***

A few of the participants referred to the prison library as a source of information. Some also said that they could talk to health care professionals, correctional officers, or other staff members like the priest, to get mental health information. Fellow incarcerated individuals who had experienced mental health problems and received health services were also mentioned by some participants. The common thread in all suggestions was a dependency on others to access information about mental health. Only a couple of participants had tried to find mental health information during their time in prison. However, they found it difficult to obtain:

*The only choice I have is to ask the prison officers to print it [mental health information], but sometimes they don’t want to do it because they think it’s bad. And I have tried to search for psychosis and such in school [in prison], but then the teachers ask why I would seek out such a gloomy subject. It feels a bit complicated to obtain information.* ***Larry***

Participants from all three prisons also pointed out the need for more information about mental healthcare in prison:

*We have a notice board on the ward (…). The information should be hung there for people to see, that there is a psychologist here, and that you can talk to her. ‘cause I’ve seen little of that sort in here.* ***Liam***

One participant underscored that information about available mental health services is particularly important for those with no experience from such services prior to imprisonment:

*It [information] must tell you about your opportunities. To normalize it [seeking help] in a way. And the threshold must be low. I think many experience that it is too high. If I hadn’t been in contact with mental health services before I came here, the threshold for seeking help would have been sky high for me as well.* ***Neil***

#### Awareness of mental health issues

Factors in the prison context were fundamental to the participants’ explanations of mental health problems. Many participants attributed the onset or worsening of mental health problems to the shock of imprisonment and to the continuous hardships of prison life. Understanding symptoms as primarily caused by external stressors such as prison hardship may have influenced their appraisals about the need to seek help. As Frank stated:

*I’ve always had good mental health. Until I came here, inside these walls.* ***Frank***

Frank reported considerable symptoms of post-traumatic stress. Understanding his symptoms as something triggered by the prison living conditions, he did not see how seeking professional help could benefit him. Like many other participants, he insisted that the correctional system needed to change and had lost hope that he could improve his own situation.

In contrast, other participants who attributed their mental health problems to external stressors concluded that they indeed needed help to cope. The suffering they experienced during their first weeks in prison motivated them to seek formal help:

*I asked to talk to a psychologist in here. ‘Cause, I felt that I needed to. ‘Cause in the beginning when I came here, it all seemed dark. No matter how hard I tried to do the right thing, there was some sort of dark force that was just pushing on, and the obstacles were piling up.* ***Travis***

For some, their main motivation for seeking help was to receive professional validation from healthcare personnel regarding the negative health consequences of their prison experiences. Some also hoped that healthcare professionals could advocate for better living conditions:

*And it is good that others [psychologists] can take part in these things. So that it is manifested what prisons actually do to people.* ***Jack***

### Social influences on help-seeking

#### Prison culture and mental health stigma

The participants described how the culture within prison influenced their willingness to talk about mental health issues. The importance of appearing strong and dominant within the prison setting was emphasized by many. According to several participants, the talk at the wards was characterized by attempts to one-up the others’ stories about criminal activities to appear tough. Many also explained that hiding vulnerabilities was critical in the prison community, and some also underlined the potential for victimization for those who were not able to conform to the prison norms:

*You are wearing a prison mask. You cannot show weakness. ‘Cause then you’ll soon be a victim, a sitting duck. I have experienced inmates that have, eh mostly stayed in their cells. They have been harassed so badly that they are sitting there crying. The prison milieu can be tough.* ***Neil***

Choosing to confide in and seek advice from peers can also have negative consequences. Several of the participants said that it was wise to be careful with who you chose to share mental health related issues with:

*Let’s say you talk about your personal feelings, and about your sentence and stuff, right. They can be very nice to you there and then, before they stab you in the back later on, spreading everything you’ve said to destroy you. It is a cynical game.* ***Bobby***

Bobby went on to explain that a fellow incarcerated individual could use personal information for harassing, blackmailing and threatening the family of someone who has confided in them, if a conflict should arise. Some of the participants also addressed directly how the prison climate may influence willingness to seek mental health treatment:

*They do not want to go to a psychologist and talk. Because then they are seen as weak and not able to cope. Because in prison everyone should be tough. Drug lords and such. But, on the inside they are not like that.* ***Nicky***

### *The role of peers in accessing mental health services*

Despite the clear barriers, fellow incarcerated appeared to be an important informal help source for mental health problems. Many of the participants had observed signs of emotional distress among their incarcerated peers and described how they had given them advice and encouragement. According to several participants, those imprisoned also had an essential role in recognizing mental health problems in their peers:

*There is no-one who talks to us regularly to check on how we are doing. That’s not a priority here. So, unless some of the inmates take on the role of an officer or a psychologist, then there’s no-one who reports concern (…) There are many inmates who are taking on a role as a social worker, but it’s kinda wrong. They are neither paid for it, nor qualified. They do it because no-one else does.* ***Stuart***

Although none of the participants said that they themselves had been prompted to seek help by peers, they told stories of how they had pushed their peers to seek formal help:

*A fellow inmate. I could tell he was struggling because he talked to me as the only person. In a way, I was his psychologist. The days when he was down in the dumps, I tried to talk to him (…) And I said, listen up. It’s for your own good. I will write a request form, and we will arrange contact with a psychologist (…) and it will help.* ***Nicky***

Experiencing fellow incarcerated people in distress appeared to be common, and participants also explained how they reported to prison officers their concerns about peers with self-harm and suicide plans:

*There was a fella’ who told me that he knew exactly how to take his own life (…). “I’ll just do it like this and this and this”. And, uhm. Then he said he was going to do it. And I thought that I would have to report it, and I did.* ***Roy***

Roy went on to describe in detail how his reported concern led to a prison officer interrupting the suicide attempt by the fellow incarcerated, thereby saving his life. Several other participants shared similar stories, indicating that peers played a significant role in recognizing and getting help for mental health related problems in prison.

### Access to mental healthcare

#### Self-referral and disempowerment

In order to access prison healthcare, those imprisoned must write and deliver a paper-based request form. All the participants in this study were aware that this is the way to contact prison healthcare, and most of them knew that the general practitioner working at the prison could refer them to a psychologist or to a psychiatric hospital. Unfortunately, the request form system seemed to amplify the participants’ perceptions of disempowerment. Rather than seeing themselves as agents taking charge of their own situation and health, they were left passively waiting to be contacted after filling out the forms:

*You are pacified when you must write a request form to talk to someone. Then you don’t know when they are coming to talk to you. And then it’s like, the problem may be swept under the rug when they finally get to you.* ***Tommy***

According to the participants, many of these request forms seemed to disappear, and it could take an exceedingly long time before they got any response to their request:

*Many times, when you write a request form it disappears. Nothing happens. Those request forms are worthless most of the time.* ***Keith***

There were also several participants who voiced concern over the confidentiality of the request forms even when the forms were delivered in closed envelopes:

*We can see for ourselves that they [prison officers] open and read, uhm, confidential information, [lowers his voice] and to put it mildly, uhm, breaches in confidentiality are all too common. It is alarming!* ***Neil***

One of the informants also explained that incarcerated persons who had mother tongues other than Norwegian could have problems with understanding and filling out request forms to health, and that forms that were not filled out correctly were of no value. According to Roy and other participants, the correctional system did not give sufficient information and guidance about the request forms:

*They might not know how to write, or understand what it [the form] says, you know? Potentially it is severe for that guy, right. It’s garbage! Garbage, that request form. They haven’t received any request from him.* ***Roy***

### *The perceived availability of mental healthcare*

The perceptions of accessibility of mental health care varied between the participants. A few of the participants were in active treatment with a psychologist at the time of the interviews, and they had experienced the access as unproblematic. Common for some of these participants was that they had been in treatment before they entered prison:

*From sending my request and to receiving an acceptance letter it took one and a half weeks. Less than three weeks later I was in treatment. It was efficient. Much quicker than I’ve ever experienced before.* ***Neil***

However, many participants said that they could not access secondary mental health services. There were two notable sub-groups among the participants who perceived that access to specialized psychological treatment was limited. The first group shared stories about living unstructured lives at the edge of society. They seemed to have little confidence in health care and correctional services, and were less hopeful of their own potential of being rehabilitated:

*I have tried for several years now, but I didn’t get help. They can say whatever they want about how easy it is to access a psychologist and prison healthcare and everything, but it is not true.* ***Ronny***

Two of the participants explained how they would have to take drastic measures such as performing violent acts or acting weird to get help for their mental health problems When Marlon was asked how he could access mental health services he responded:

*You would have to either hurt yourself, or someone else, so that they end up in hospital.* ***Marlon***

The interviewer asked if it was possible to access mental health services by using less drastic measures, Marlon answered:

*Uhm. Naaah. I don’t know. I do not think so. Not from my experience.* ***Marlon***

Another sub-group having difficulties accessing mental health services was those in prison for the first time. Most had led more typical lives with stable employment and housing conditions before imprisonment. When they sought mental health services, they were told that these adjustment problems were normal in prison:

*I’ve been struggling for several periods here and have said that I wanted to talk to a nurse or a psychologist. And then I was referred to a psychologist. And the psychologist assessed me, and said that: “Nothing’s wrong with you, you are just having a hard time, I cannot help you”. So, you do not get anyone to talk to, unless you- I don’t know what you must have really, but I sure ain’t got it. The nurses say that they haven’t got the time, and the psychologist says that I am not ill. And then I am left to feel bad. In my case, there is no service really.* ***Stuart***

#### Prison officers’ role in mental healthcare and accessing services

Several participants stated that mental health problems and well-being were not high on the prison agenda. Many would have appreciated it if correctional officers on a more regular basis had asked how they were doing and believed that this would have facilitated them to open up and talk about mental health issues.

*In my opinion, mental health is forgotten here in a way. Physical activity, movement, workouts, yes. Since I arrived here some months ago, only twice I’ve been asked: “Hi, how are you? Is there something you want to talk about?”* ***Travis***

Some also said that they knew people in prison who were unaware of their own need for mental health care or unable to access help, and argued that the correctional system should do more to help these people to access care:

*You have the type where people do not get help because they themselves are not able to request help from the prison health services and the prison officers do not see to that they get the help they need.* ***Neil***

Some were concerned about how acute health problems were handled in the weekends and evenings when prison health services were unavailable. In these situations, prison officers were left to decide whether or not to contact emergency healthcare services. Several of the participants were not satisfied by this arrangement:

*(…) they think that they can make a doctor’s judgement. That they can decide that it is not that important. It is rude. It is trespassing norms.* ***Jack***

Some participants told stories of how their peers in prison did not seem to get the help they needed even though it was apparent that they were in a bad state mentally:

I have reported concern about people, before they started cutting themselves and f***ing themselves up. But what worries me, is that even though I voiced my concern to both prison health services and prison officers, no measures were taken. Before it was too late. ***Stuart***

Asking for help from correctional officers could also have consequences. Ronny served at a lower security level. He experienced that his requests to see a psychologist were met by suggestions of transferring him to a higher security level:

*I have written request forms: “I need to speak to a psychologist. Immediately”. And then they [the prison officers] are threatening me by saying that they are going to transfer me to a higher security level. They ask if I am going to hurt myself. No, I tell them. I’m not going to hurt myself. I just need to talk to a psychologist.* ***Ronny***

Another participant described how he had sometimes cut himself by shards from plates and drinking glass to suppress mental suffering. He explained how he on one occasion used the intercom to notify the officers that they needed to come and pick up a glass that was triggering an urge to self-harm. The participant said that initially a single officer came to his cell to pick up the glass:

*A few minutes later there were four officers, and they unlocked the cell door, and there were a lot of questions. I guess they were worried about my mental state, and I said that I appreciated the concern. Then I reminded them that I had asked them to pick up the glass so I would NOT cut myself, so if they were to use that against me, it would be unfair.* ***Tommy***

He reassured that the situation had been resolved with the conversation. However, he had the impression that disclosing mental distress to officers could increase the risk for being transferred to a higher security level, or to a security cell.

### Enhancing access to services

#### The perceived advantages of seeking professional help

There were some commonly experienced benefits of seeking mental healthcare among the participants. Coming off drugs and living under stable conditions in prison provided some participants an opportunity to reflect on their lives and to gather motivation to work on their addiction and mental health problems:

*I have been thinking a lot about treatment in an institution. I know how it went the last few times I got out* [of prison]. *Within half an hour I was sitting there with the needle. And if I don’t do anything before I get out, the same will happen again. I’m trying to prevent it (…) I’ve had treatment for drug and alcohol use before. And back then there was a psychologist who said that, once you’ve been clean for a year, then the brain is back to normal. I can feel it, like, my mindset is already changing*. ***Kurt***

For about half of the participants, seeking professional help was related to their motivation for living a law-abiding life after prison. The participants linked substance use to both mental health problems and a criminal lifestyle, and getting treatment was seen as essential for preventing recidivism:

*I have lived a rough life, and I have no-one, NO-ONE. How long am I going to live? One doesn’t know. But I’ll be fifty soon. So, I must make it now. I really have to make it now [his voice bursts]. And it depends on many psychological factors. So, I’m choosing to use all the things that I have access to in prison, like treatment for drug addiction.* ***Roy***

Although many had previous experience of treatment for their substance use, they still had hopes that treatment could help them. Liam had previously experienced that consultations with a psychologist brought up subjects that was difficult for him to talk about:

*I regret that I quit, because it could have done me good. But I guess it got too personal, and it stirred up things.* ***Liam***

He also explained that at the time he was more interested in doing drugs than going to therapy. However, he still believed that treatment could help him:

*I will probably contact a psychologist, now that I’m about to get treatment for my addictions. It is easier to open up when there are no substances involved.* ***Liam***

In summary, seeking professional help for mental health problems was perceived to promote in-prison coping, rehabilitation, and preparation for life outside of prison for most of the participants.

***Lowering the bar for accessing mental health services***.

Many of the participants expressed skepticism towards ‘the system’. They described how they had been let down and disappointed by the child welfare services, the criminal justice system, and healthcare professionals. Experiences from childhood to adult life had led to a lack of confidence that healthcare personnel and the correctional system and society had their best interest at heart. For them, it was important that healthcare professionals were perceived as genuine and “on their side”:

*The experience of being believed and listened to… They do not have to relate, to say that they understand so damn much, ‘cause that’s not really important.* ***Marlon***

Several participants said that barriers for talking about mental health were reduced when healthcare personnel reached out in the prison ward. One of the prisoners described two nurses who used to visit the prison wing every day at lunch-hour. He appreciated that it was possible to request a private conversation in the cell, and that he was taken seriously:

*They were highly skilled. And they listened. They listened to what you had to say, and they understood you.* ***Tommy***

Having previous positive experiences of mental health treatment and knowledge of what to expect from mental health services also seemed to reduce barriers for in-prison help-seeking from some of the participants:

*I saw a psychologist on a regular basis, once a week (…). And after six consultations I was past the worst in some sense. I was provided with the tools I needed to cope. ****Bobby***.

This participant had experience with psychological treatment outside of prison and had tried to access mental health services for months in prison. However, he believed his challenges were too mild to get help from a psychologist. He emphasized the need for available low-threshold services for those who suffer from milder mental health problems:

*It should be available for everyone who wants it. It should not be embarrassing, it should not be taboo, it should be… A natural part of it, really.* ***Bobby***

In addition, when services were provided as standard procedure and a natural part of rehabilitation, they were perceived as less stigmatizing. Nicky described how he was placed on a prison ward that was specialized in substance use treatment:

*And when you are placed in that ward, then you are automatically assigned to a psychologist from the substance use clinic, that you can have weekly consultations with. ****Nicky***.

Some also suggested that the systematic screening and assessment of health and social problems also could facilitate access to mental health services and this was suggested as an integral part of healthcare and rehabilitation in prison by some of the participants. Ronny underscored the importance of proper assessment:

*What is this person’s problem? Why did he come back? Is there something happening to him on the outside? Could he need help with anything? Maybe someone should ask him?* ***Ronny***

Ronny went on describing the nice brochures of the correctional system, with promises of assessment of strengths and needs of individuals, but he claimed that this did not happen in reality. This view was shared by several of the other participants, as they called for more assessment to benefit the mental health and rehabilitation of incarcerated individuals.

***Mental health support from different sources***

The participants had different preferences regarding where to get help. Support from friends and family was seen as important for most of the participants. However, health professionals could sometimes be preferred over informal or semi-formal sources because of their role in advocating for better living conditions in prison:

*I get visits from my family, but I’d like to talk to someone here in prison, so that they could gain awareness of the actual problem. If I’m spitting venom to some random lady that is here as a volunteer with the Red Cross, it’s useless, I think. If I talk to a nurse who works here at this establishment, she could perhaps do something about some of our challenges.* ***Stuart***

The cultural competency of health care personnel could also be a key factor in promoting help-seeking and forming a therapeutic alliance with people in prison. Many incarcerated individuals have lived on the edge of society, while most health care personnel, and particularly doctors and psychologists, are from the upper middle class. These cultural differences may form an abyss between the incarcerated individuals and mental healthcare personnel:

*A psychologist does not have a criminal record. Now I’m generalizing. But they have performed well in school. Have passed through the system. Highly educated. Their lives have been smooth sailing (…) They have not experienced the shadow side of life.* ***Tommy***

This participant had one prior positive experience with a psychologist, but his general impression of psychologists was that they were of no help. He did not feel a connection with any of the others and had written them off completely. He preferred talking to a representative from a user organization who have led a similar life to himself:

*I know that they know exactly how I’m feeling. They have served a prison sentence. And they… They have lived experience, and then it’s much easier to listen to what they have to say, because I know it’s not knowledge that they have acquired through reading.* ***Tommy***

Prison officers can also be of help to incarcerated people who experience mental health problems. Nicky said that while he was at a lower security level, he had been to a sports event outside prison with an officer and some fellow incarcerated. He had a panic attack because of all the people who kept arriving at the venue and he had to go outside for some fresh air. The prison officer followed him and was understanding, and told Nicky that he had seen many incarcerated people with similar reactions:

*He was understanding and said: It will be OK. After that day at the match, coincidently, he ended up being my primary contact officer. And to socialize me back to society he fixed it so that every weekend he was working we could go to a shopping mall, to try. Little by little, by little. (…) It helped. It did. Yes.* ***Nicky***

Although Nicky had no plan to seek help for his anxiety symptoms, he appreciated the support he received from his primary contact officer.

Bobby, on the other hand, had some informal support from fellow incarcerated and had also talked with a priest. He said that he often ruminated when he had time alone in his cell and emphasized his need for sharing his thoughts with others and receiving advice. He explained why he preferred to get help from formal sources:

*So, to have someone who is an outsider. Who’s not an inmate. Who has got a sensible outlook on life, that can guide you– I think that’s important. (…) Because when you talk to a fellow inmate, then… It can go in the opposite direction, right. Because many have been through major crises, they have lost friends, they have lost family, maybe they have lost their girlfriend and wife, their children won’t speak to them, right?* ***Bobby***

Most participants also held the prison priests in high regard and appreciated the availability of the service. However, talking with a priest was not seen as a replacement for a consultation with a psychologist:

*It was peculiar, when I asked for someone to talk with, the priest was offered first. For me it is alright, I go to church. But I’m thinking, if someone is not a Christian. I’m like: a priest? Or if you’re not religious. A session with the priest is more like a consultation towards God and his will. He can be a good listener [the priest], but you might not get the help you need in a mental sense. So, a psychologist, a “talking person” in prison is necessary. That could check on you sometimes.*

**Travis**

## Discussion

This study’s findings demonstrate that many of the factors deciding access to mental healthcare are firmly rooted at the organizational level of the correctional and healthcare systems. Prisons in the Scandinavian countries, including Norway, are presumptuously humane compared to harsher correctional settings in other parts of the world. One could assume that these favorable conditions would be more conducive to mental healthcare access. However, the systemic barriers we found largely overlapped with challenges reported in other countries [42–44]. In addition, we found that individual beliefs, attitudes and aspirations also influence willingness to seek mental healthcare. Interestingly, most of these intrapersonal factors are tightly interwoven with the participant’s appraisals of how the prison conditions influence their mental health. This study also addresses an important knowledge gap in the literature, namely how restrictions on access to mental health information could influence mental health help-seeking for people in prison. The identified core category, “Breaking down barriers”, reflects an overarching focus on solutions to improving mental healthcare access based on the experiences of the participants in this study.

### Access to health information

Knowledge of available services and how to access them is a prerequisite for mental health help-seeking [80]. The participants in our study claimed that information about mental health services was unsatisfactory, and lack of such information has also been noted as a barrier to help-seeking in other prison-based studies [44]. Moreover, sufficient levels of mental health literacy are positively associated with increased intentions for help-seeking from both informal and formal sources [81]. The participants in our study reported severely restricted access to their preferred sources of health information and a dependency on others to obtain such information. Since information seeking may occur before individuals are ready to share their health concerns with others, having to rely on others for accessing information is a potential barrier for recognizing mental health problems [25]. Thus, it is likely that the limited access to mental health information negatively impacts incarcerated persons capacity to manage their own mental health needs. The potential consequences of restrictions on access to health information among people in prison need more research attention. However, findings from other populations suggest that closing the apparent health information gap could be an important intervention for improving help-seeking for mental health problems [82, 83].

### The social influences on accessing mental healthcare

The participants reported that prison culture reduced their willingness to seek support from fellow incarcerated and the use of professional help for mental health problems. The TBP element “subjective norms” posits that beliefs about the opinions of others may influence the willingness to seek help [29]. Attributing mental health problems to personal weakness may reflect a stereotyped attitude involved in stigmatizing mental disorders [84]. Stigma may lead to concerns about what others might think if one were to seek help, and may delay or hinder help-seeking efforts [80, 85]. It also seemed to be an important constraint to mental healthcare access in our study. This corresponds with findings from other studies [45–47] and suggests that fear of appearing weak is also a significant barrier to help-seeking in a Scandinavian prison context. Based on our findings and recommendations, we advise that focus on health education and normalization of mental health problems are measures that could decrease stigma [86], and increase willingness to seek mental health support and treatment among people in prison.

Although the culture among those incarcerated was perceived to discourage seeking support for mental health problems, fellow incarcerated also played a key role in supporting those who experienced mental health problems. They were more available than other help sources and had lived experience with distress related to imprisonment. Since information about available services was insufficient, fellow incarcerated were also perceived as an important source of information. Thus, naturally occurring peer support seemed to normalize mental health problems, possibly reducing stigma and lowering the threshold for mental health help-seeking. From the literature, we know that peer-based health interventions is effective in correctional settings [87], and formalizing peer-based health information and support could be beneficial in interventions aiming to increase the use of mental health services.

### Beliefs and motivations for help-seeking

The prison environment was embedded in the participants’ beliefs: attributing the onset and worsening of mental health problems to the prison conditions was common among the participants. According to the Theory of Planned Behavior (TPB), attitudes about the potential benefits of help-seeking and alignment with individual goals affect the readiness and willingness to seek professional help [29]. Our data supported this notion. Some participants abstained from seeking professional help as they did not see how it might benefit them in their goal of improving their living conditions. For others, a prominent motive for seeking professional help was to receive validation and help managing their challenging life situations and the everyday stressors of prison life. A few participants also framed mental health help-seeking as a mission to document the consequences of imprisonment. By sharing their experiences with professionals, they hoped healthcare personnel could help them advocate for better conditions in prison. Obtaining sufficient knowledge about essential aspects of prison life is essential for health professionals working in a prison setting [88]. Based on our findings we propose that the ability of healthcare staff to communicate their understanding of the influence of prison living conditions on mental health is crucial for gaining trust and building an alliance with their incarcerated patients.

Another important motivator for many participants seeking help was their aspirations to live a law-abiding life after being released. It has been increasingly recognized that the relationship between mental disorders and criminal activity is complex and that integrated treatment that addresses both criminogenic factors (i.e. antisocial attitudes and behavior, substance use, criminal network, family issues and low educational/vocational engagement) and mental health issues is a must to prevent recidivism [89]. This view corresponds with the beliefs and preferences for rehabilitation and healthcare of several participants in our study. They were worried about their reintegration into society, which motivated them to seek professional help. Substance use treatment, in particular, was seen as essential to attaining rehabilitative goals. However, some participants who had served multiple sentences were less positive towards help-seeking. They had more negative experiences and seemed less hopeful that mental healthcare could improve their situation. Their low expectations for potential gain combined with a perceived lack of personal control in the help-seeking process, appeared to stall help-seeking for these participants. We suggest that implementing health services with a concurrent focus on addressing both criminogenic needs and mental disorders could be especially important for fostering healthcare utilization for people with a history of reoffending.

### Organizational barriers to accessing mental healthcare

The perceived challenges with the paper-based request system were considered a significant barrier to healthcare access. TPB postulates that behavioral control and self-efficacy are important in help-seeking [29]. In a system where autonomy is limited, one could assume that a self-referral system can be empowering for those seeking help. However, the participants seemed to experience the opposite as they were left passively waiting for an answer to their request. Some also expressed confidentiality concerns, as they believed that prison officers read the request notes. Thus, the process of accessing health services seemed to diminish, rather than enhance their notions of control and self-efficacy. Improving the reliability of responses to requests and ensuring confidentiality could increase the experience of control in the self-referral process and may also empower imprisoned persons to seek help.

A barrier rooted in the interactions between the individual and the helping services was found in various expressions of skepticism towards “the system” by many participants. Earlier studies have also reported distrust in the system as a barrier to help-seeking [41, 44]. Our results elaborate on these findings as the participants spoke of how suicides and severe self-harm by fellow incarcerated people contributed to diminished faith in the system. Some had voiced concern over the health and welfare of peers and had experienced that they were not listened to by the prison officers. According to the participants, many of their fellow incarcerated people had more severe symptoms of mental health problems and did not seem to have access to the help they needed. This confirmed their beliefs that the system took little interest in their mental health, and for some of them this led to a growing feeling of hopelessness and resentment. In addition, the high prevalence of mental disorders in prison implies that incarcerated persons witness people in severe distress regularly and for prolonged periods. This issue is largely unexplored and unrecognized in prison research, and the impact of these experiences on mental well-being and recovery should be investigated further.

Participants who experienced mental distress and adjustment problems had difficulties in accessing mental health services. They needed someone to talk to about their situation that could give them advice on how to cope, however they did not fulfil the criteria for secondary mental health services. Minor mental health problems in Norwegian prisons are to be handled by the prison healthcare services. However, according to the participants their capacity is very limited. This finding corresponds to other studies [90] documenting that access to integrated mental health services was limited for those with milder mental health problems. In the community, the establishment of low threshold services for people with mental health problems has been an important commitment as early intervention can prevent the development of more serious conditions. This may be even more important for those imprisoned, since coping strategies such as physical activity and seeking social support are less accessible [91].

### Prison officer’s influence on access to mental healthcare

Prison officers were perceived to have a key role as gatekeepers to healthcare. Officers can facilitate access to healthcare by encouraging help-seeking or directly contacting healthcare services based on observations and conversations with incarcerated individuals [39, 41, 92]. The participants in our study pointed out the need for prison officers to take their health concerns more seriously, and that the threshold for contacting healthcare services by their request was too high. In addition, being asked directly about their psychological state by staff members was seen to ease talks about mental health by the participants. Our results support the notion that prison officers that are responsive to the mental healthcare needs of incarcerated persons could build confidence that these needs would be attended to when required [92]. Thus, ensuring sufficient mental health knowledge and awareness among prison officers of their role in mental healthcare access is an essential task for correctional systems.

Previous studies have found that the correctional systems´ procedures for managing suicidal risk is a potential obstacle for help-seeking. The fear of being moved or placed in a safety cell without personal belongings was identified as a barrier to disclosing suicidal thoughts [39, 93]. In Norway, the risk of self-harm and suicide is ideally handled by increasing social contact, activities, monitoring and healthcare. However, in the face of acute mental crisis and severe suicide risk, placing persons in solitary confinement is not an uncommon practice [94]. Challenges with having incarcerated persons admitted and treated in specialized health care institutions, understaffing, and a lack of central guidelines for handling suicide risk may contribute to the use of solitary confinement for incarcerated persons in acute mental distress in the Norwegian correctional system [94]. The Norwegian Parliamentary Ombudsman reports that fear of solitary confinement and being placed in a security cell is a barrier to seeking help for suicidal ideations and plans [95]. In our study, participants who had asked for help when they were in acute distress experienced that the officers assumed that they intended to harm themselves. They were faced with the potential of being transferred to a higher security level or being placed in solitary confinement. Thus, how prison officers respond to incarcerated persons’ reports of acute mental distress could be of critical importance for their willingness to seek help for mental health issues in the future. However, more research on the perceived and actual consequences of disclosing mental distress and suicidal ideations in prison is needed to inform interventions to promote help-seeking.

### Enhancing access to mental healthcare in prison

The participants underscore some conditions that may lower the bar mental healthcare utilization. Earlier positive experiences with mental healthcare in the community was mentioned by participants as important for their willingness to seek such services in prison, which also corresponds with findings in earlier studies [42, 96]. In addition, the participants saw mental health services that were outreaching and integrated as positive. A few participants also highlighted mental health screening at reception to discover mental disorders that may need intervention. Screening at intake, and outreaching and integrated services are also recommended in the prison research literature [88]. Our findings show that these recommended measures may also make intuitive sense to incarcerated persons - common for all of them are that they seem to reduce stigma related to utilizing mental healthcare.

Our results indicate that incarcerated persons with both milder and more severe mental disorders experience barriers to accessing mental healthcare. These results are in line with studies from other correctional settings reporting unmet needs due to challenges with access and delivery of mental healthcare [37–39]. The underutilization of mental health services by incarcerated persons suggests that the ‘degree of fit’ between their needs and the available mental healthcare requires improvement. The World Health Organization (WHO) advocates for correctional systems with health and well-being as an integrated part of their core business and culture [33]. Along these lines, we found that participants called for a correctional system with mental health higher on the agenda. Some also preferred to seek help for mental health problems from other sources than mental health professionals. This finding supports the recommendation of the WHO that it is important to build mental health competency in all staff members in contact with those imprisoned. As many of the barriers to mental healthcare utilization are rooted in the wider correctional setting, we also suggest that the correctional and healthcare systems, in collaboration, should review their practices to enhance perceived efficacy in accessing healthcare.

### Limitations

The data in this study are based on interviews with fifteen participants from three prisons. The participants were self-selected and may have had more knowledge, interest, and willingness to talk about mental health issues than the average person in prison. We cannot claim that the results represent a complete account of access to mental healthcare and help-seeking among incarcerated persons in Norway. However, our findings were consistent with findings from other studies from Norway and correctional settings in some other countries. We have presented details about the participants, method, data, and context to allow others to consider the potential transferability of the results. We hope our findings encourage further research on access to mental healthcare for people in prison.

## Conclusions

Mental healthcare that is outreaching and integrated is perceived to facilitate access and decrease stigma. The correctional system should address access to health information, the referral system, and their responses to incarcerated persons who disclose distress to facilitate access to healthcare. Our results also indicate that mental healthcare extends beyond the scope of health services, suggesting that sufficient mental health knowledge and agency is needed at all levels of the correctional system.

## Data Availability

The data produced in the course of this research is not openly accessible owing to considerations regarding privacy. However, they can be obtained from the corresponding author upon a reasonable request.

## Abbreviations

TBP: Theory of planned behavior
MHL: Mental health literacy
